# Ringworm in the Light of Recent Research

**Published:** 1898-12

**Authors:** 


**Ringworm in the Light of Recent Research.** By MALCOLM MORRIS. Pp. viii., 142. London: Cassell and Company, Limited. 1898.

All the new views about this troublesome complaint, as well as a very concise account of its earlier history, are to be found in this volume. Sabouraud's investigations and theories are dealt with, and no credit due to him is withheld. There is no question whatever that Gruby more than fifty-five years ago discovered the "microsporon" of Sabouraud, for Sir Erasmus Wilson, in an edition of his work published in 1842, quotes the following from Gruby (translated): "The whole of the dermic portion of the hair is surrounded by cryptogamic formations, which constitute a vegetable sheath around it, in such manner, that the hair implanted in this vegetable sheath may be likened to the finger surrounded by a glove." Sabouraud makes many species of the fungus, which Mr. Morris very well demonstrates may be reduced to two in number, having clear distinctions; viz., a small spored variety (microsporon) and a larger spored one (megalosporon). At present it seems wiser not to exceed these.

The work is illustrated with one plate—very well coloured—of the spores, and twenty-two photomicrographs very clearly delineated. The printing and setting up of the book are good. Chapter XI., on Treatment, is very comprehensive, and gives due consideration to the opinions of others; and Chapter XII., on Prophylaxis, is well worth perusal and reflection. The index merits a special word of praise.

